# A Novel Model for Predicting the Clearance of Jaundice in Patients With Biliary Atresia After Kasai Procedure

**DOI:** 10.3389/fped.2022.837247

**Published:** 2022-01-31

**Authors:** Yimao Zhang, Qi Wang, Siyu Pu, Junxiang Wang, Bo Xiang, Juxian Liu, Shuguang Jin

**Affiliations:** ^1^Department of Pediatric Surgery, West China of Hospital, Sichuan University, Chengdu, China; ^2^Department of Neonatal Surgery, Shenzhen Children's Hospital, Shenzhen, China; ^3^Department of Ultrasound, West China of Hospital, Sichuan University, Chengdu, China

**Keywords:** biliary atresia, Kasai, clearance of jaundice, nomogram, prediction model

## Abstract

**Background:**

The failed clearance of jaundice (CJ) in patients with biliary atresia (BA) after the Kasai procedure (KP) often leads to a shorter native liver survival (NLS) time and earlier liver transplantation. We aimed to investigate risk factors of failed CJ and establish a novel nomogram model to predict the status of CJ.

**Methods:**

We retrospectively reviewed institutional medical records from January 2015 to April 2020 and enrolled BA patients post-KP, randomly divided into training and testing cohorts at a ratio of 7:3, and further subdivided into cleared and uncleared jaundice groups. Univariate and multiple logistic regression analyses were used to select risk factors to establish the nomogram in the training cohort. The performance of the nomogram was evaluated by calculating the areas under the receiver operating curve (AUC) in both cohorts.

**Results:**

This study included 175 BA patients post-KP. After univariate and multiple logistic regression analyses, Cytomegalovirus IgM +ve associated BA (OR = 3.38; 95% CI 1.01–11.32; *P* = 0.04), ln γ-glutamyl transpeptidase (GGT) (OR = 0.41; 95% CI 0.22–0.80; *P* = 0.009), thickness of the fibrous portal plate (OR = 0.45; 95% CI 0.27–0.76; *P* = 0.003), liver stiffness measurement (LSM) (OR = 1.19; 95% CI 1.06–1.34; *P* = 0.002), and multiple episodes of cholangitis (OR = 1.65; 95% CI 1.13–2.41; *P* = 0.01) were identified as independent risk factors of unsuccessful CJ to construct the nomogram. The receiver operating characteristic curve (ROC) analysis suggested good nomogram performance in both the training (AUC = 0.96) and testing cohorts (AUC = 0.91).

**Conclusion:**

Our nomogram model including several risk factors effectively predicts CJ in patients post-KP, which could aid in clinical decision-making.

## Introduction

Biliary atresia (BA) is a rare and severe cholangiopathy that leads to liver failure in infants and is characterized by a progressive fibro-inflammatory process affecting the intrahepatic and extrahepatic bile ducts (1). The pathogenesis of BA remains unclear, and the majority of untreated patients die before the age of 1 year due to liver failure (2–4). Five- and ten-year native liver survival (NLS) rates in BA patients have been documented as 46–58% and 30–40%, respectively (5–8). Despite advances in surgical techniques and perioperative management, liver transplantation is required for these patients to achieve long-term survival (9).

Some studies have indicated that a rapid, early, and complete clearance of jaundice (CJ) after the Kasai procedure (KP) was the most valuable prognostic factor for long-term NLS (10, 11). Moreover, unsuccessful CJ in BA patients was always associated with a shorter NLS and an earlier time for pediatric liver transplantation (12). Several studies reported that the post-KP CJ widely varied from 36 to 61% (3, 8, 13). Although previous studies have identified some clinical risk factors for unsuccessful CJ (14–16), it is still difficult to make an early and precise prediction of CJ in clinical practice. However, an early and precise prediction of CJ is essential for clinicians to asses risk stratification, estimate the prognosis, develop individualized treatment, and prepare for further liver transplantation, such as ethics application, implementation of intensified nutritional support, and monitoring of potential complications related to progressive liver disease (17, 18). We hypothesized that a visual prediction model based on clinical factors can accurately predict the CJ. Therefore, the purpose of this study was to investigate risk factors of failed CJ and establish a novel nomogram model based on these selected factors to predict the CJ in BA patients post-KP.

## Patients and Methods

### Patients

**Figure 1:**
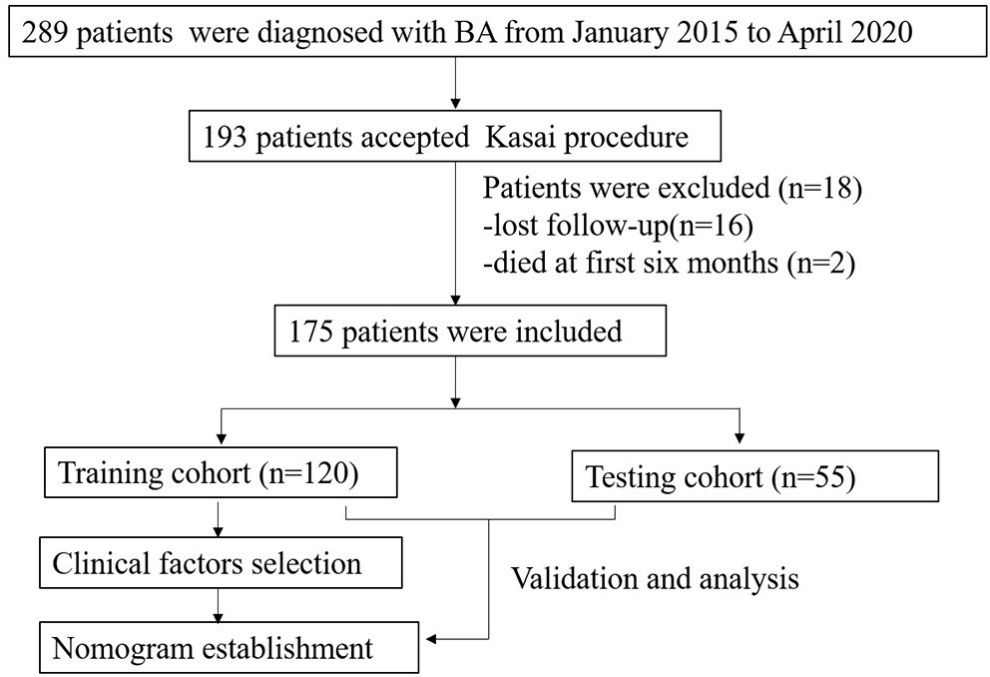
The work flow of this study.

This retrospective study was approved by the Ethical Committee of the West China Hospital of Sichuan University (ChiCTR1800017017). From January 2015 to April 2020, 289 patients who underwent intraoperative cholangiography and liver biopsy were diagnosed with BA. Patients who underwent the KP were included in this study. In all patients, methylprednisolone was administered intravenously 5 days postoperatively at a dose of 4- mg/kg/day initially, reduced by 1 mg/kg/day every 3 days for 2 weeks or even longer until the normal value of total bilirubin reached. Cephalosporin antibiotics were orally taken alternately weekly until 1 year of age. Ursodeoxycholic acid and hepatoprotective tablets were used until 3 years of age. Patients with incomplete clinical data, such as those who died within the first 6 months post-operation and those lost to follow-up, were excluded from the study. The workflow of this study is illustrated in Figure 1. Enrolled patients with cleared and uncleared jaundice were randomly divided into training and testing cohorts at a ratio of 7:3 by random number. Additionally, patients were divided into cleared and uncleared jaundice groups according to their achievement of CJ. CJ was defined as a reduction in total bilirubin (TBIL) level to <20 μmol/L at 6 months post-KP (19).

Clinical data were collected from each patient's medical records, including age at surgery, sex, weight, surgical method used, other malformations, blood loss, Davenport classification (1), Metavir scores, and times of postoperative cholangitis. CMV (cytomegalovirus) IgM +ve associated BA was determined based on the CMV-specific IgM results, which are immunoenzymatic assays based on the ELISA technique. Metavir scores were estimated from the results of the liver biopsy (20). Cholangitis was defined as unexplained fever (>38°C), reappearance of white stools, increase in TBIL levels, and increase in inflammatory parameters. The multiple episodes of cholangitis were calculated from the first day after surgery to 3 months postoperatively. An episode of cholangitis was defined as the time from diagnosis to discharge from the hospital. Preoperative liver function and coagulation results, including TBIL, alanine aminotransferase (ALT), aspartate aminotransferase (AST), serum albumin, alkaline phosphatase (ALP), total bile acid (TBA), and γ-glutamyl transpeptidase (GGT) levels, international normalized ratio (INR), and prothrombin time (PT), were also collected. The preoperative thickness of the fibrous portal plate and liver stiffness measurement (LSM) values were examined by a pediatric sonographer with 20 years of experience. Thickness of the fibrous portal plate was defined as the thickness of the echogenic anterior wall of the anterior branch of the right portal vein just distal to the right portal vein in a transverse oblique plane (21). LSM was measurement by shear wave elastography (SWE) technique. More than five valid measurements were recorded for each patient, and the median value was considered as the ultimate LSM value in each individual.

### Establishment and Evaluating Performance of the Nomogram

A univariate analysis was performed to identify significant (*P* < 0.05) clinical factors in the training cohort to establish a multivariate logistic regression model. Clinical factors with a *P* < 0.05 in the logistic regression model were used to establish the nomogram, and the predictive performance of the nomogram was evaluated by calculating the area under the receiver operating curve (AUC) in both the training and testing cohorts.

### Statistical Analysis

SPSS 26.0 statistical software and R programming (3.2 version) were used for all statistical analyses. Ln-transformation was applied to normalize the ALP and GGT values. Normally distributed data were represented as x ± s, and the skewed measurement data were represented as M. Differences were evaluated using the Student's *t*-test for continuous parametric data, Wilcoxon test for continuous non-parametric data, and Pearson's chi-squared test for non-continuous data. Statistical significance was set at *P* < 0.05. The “rms” package was used to establish the nomogram. The “pROC” package was used to plot the receiver operating characteristics (ROC) curve of the prediction model.

## Results

### Patient Characteristic

From January 2015 to April 2020, 193 BA patients underwent the KP at our center. Sixteen patients who were lost to follow-up and two patients who died in the first 6 months post-KP were excluded from this study. Therefore, 175 BA patients were included in this study. The distributions of the Davenport classification were as follows: 8 (4.6%) cases with syndromic BA, 5 (2.8%) cases with cystic BA, 48 (27.4%) cases with CMV-IgM +ve associated BA, and 114 (65.2%) cases with isolated BA. A total of 120 and 55 patients were randomly enrolled in the training and testing cohorts, respectively. The baseline characteristics of both the training and testing cohorts were presented in Table 1. There was no significant difference in age at surgery (*P* = 0.32), gender (*P* = 0.61), weight (*P* = 0.57), outcomes of CJ (*P* = 0.80), or surgical methods (*P* = 0.19).

**Table 1:** The baseline characteristics of patients in training and testing cohorts.

**Characteristics**		**Training cohort (*n* = 120)**	**Testing cohort (*n* = 55)**	***P*-value**
Age at surgery (days)		64.3 ± 17.8	65.9 ± 18.8	0.68
Gender	Male	54	27	0.61
	Female	66	28	
Weight (kg)		5.0 ± 0.9	5.1 ± 1.0	0.57
Clearance of jaundice		83	37	0.80
Surgical methods	Open	57	32	0.19
	Laparoscope	63	23	

The characteristics of the patients and univariate analysis of the training and testing cohorts were shown in Table 2. In training cohort, the univariate analysis identified significant differences in surgery days (*P* = 0.03), CMV IgM +ve associated BA (*P* < 0.001), ln (GGT) (*P* < 0.001), thickness of the fibrous portal plate (*P* < 0.001), LSM value (*P* < 0.001), Metavir score (*P* = 0.02), and multiple episodes of cholangitis (*P* < 0.001).

**Table 2:** Characteristic of the patients and univariate analysis in training and testing cohort.

	**Training cohort (*****n*** **= 120)**			**Testing cohort (*****n*** **= 55)**		
**Vriables**	**CJ group (*n* = 83)**	**UJ group (*n* = 37)**	***P*-value**	**CJ group (*n* = 37)**	**UJ group (*n* = 18)**	***P*-value**
Surgery days, mean ± SD	61.2 ± 20.0	68.2 ± 19.3	0.03	63.81 ± 18.9	69.8 ± 19.2	0.04
Male	38	16	0.79	17	10	0.50
Weight (kg), mean ± SD	5.0 ± 1.0	5.1 ± 0.6	0.55	5.1 ± 1.1	5.1 ± 0.8	0.67
CMV IgM + ve associated BA (%)	15 (18.1%)	19 (51.4%)	<0.001	6 (16.2%)	8 (44.4%)	0.02
Laparoscope Kasai	42	21	0.53	17	6	0.37
TBIL (umol/L), mean	170.5 (91.8–351.5)	181.7 (110.0–458.8)	0.84	175.8 (92.9–263.7)	189.2 (126.5–422.1)	0.69
ALT (IU/L), mean	152 (25–557)	181 (26–542)	0.50	158 (31–529)	189 (36–522)	0.45
AST (IU/L), mean	298 (100–2,228)	344 (95–1,546)	0.55	274 (103–1,980)	336 (102–1,374)	0.29
Albumin (g/L), mean ± SD	40.3 ± 3.8	40.7 ± 4.4	0.55	39.8 ± 2.9	41.4 ± 4.1	0.47
In (ALP), mean ± SD	6.3 ± 0.3	6.3 ± 0.4	0.73	6.1 ± 0.6	6.3 ± 0.5	0.12
TBA (umol/L), mean ± SD	156.0 ± 36.0	148.3 ± 38.8	0.39	148.1 ± 31.1	150.3 ± 32.4	0.64
In (GGT), mean ± SD	6.3 ± 0.8	5.7 ± 0.8	<0.001	6.2 ± 0.6	5.8 ± 0.6	<0.001
INR, mean	1.1 (0.79–4.9)	1.2 (0.84–4.08)	0.84	1.2 (0.81–3.2)	1.4 (0.94–4.01)	0.73
PT, mean	13.2 (9.5–58.6)	14.3 (10.3–44.9)	0.20	12.5 (9.8–42.5)	13.9 (10.4–51.7)	0.64
Other malformations	34	10	0.20	12	5	0.72
Thickness of fibrous portal plate (mm), mean ± SD	5.3 ± 1.2	3.9 ± 1.1	<0.001	5.5 ± 1.0	3.7 ± 0.9	<0.001
LSM (kPa), mean ± SD	11.3 ± 4.2	15.3 ± 5.4	<0.001	10.6 ± 3.9	16.3 ± 5.2	0.003
**Metavir score**						
F1–F3	50	14	0.02	21	7	0.21
F4	33	23		16	11	
Blood loss (ml), mean	24.0 (5–100)	27.4 (5–60)	0.28	26.2 (5–70)	26.8 (5–100)	0.73
Multiple episodes of cholangitis, mean	1.2 (0–4)	2.4 (0–5)	<0.001	1.1 (0–3)	2.0 (0–4)	0.03

*CJ, cleared jaundice; UJ, uncleared jaundice; CMV, cytomegalovirus; TBIL, total bilirubin; ALT, alanine aminotransferase; AST, aspartate aminotransferase; ALP, alkaline phosphatase; TBA, total bile acid; GGT, γ-glutamyl transpeptidase; INR, international normalized ratio; PT, prothrombin time; LSM, liver stiffness measurement*.

### Establishment and Validation of the Nomogram Model

**Figure 2:**
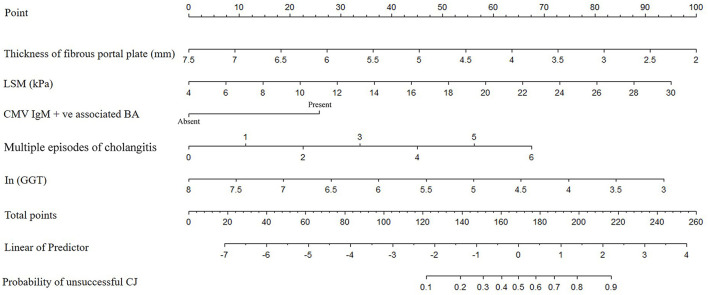
The nomogram for prediction the possibility of unsuccessful CJ in patients with KP. (Each clinical factor with different status would correspond to a score in “Point”. The scores for each clinical factor were added to obtain an overall score in “Total point”. Then, “Total point” would correspond to risk of “Probability of unsuccessful CJ”.) CMV, cytomegalovirus; GGT, γ-glutamyl transpeptidase; LSM, liver stiffness measurement; CJ, clearance of jaundice; BA, biliary atresia; KP, Kasai procedure.

**Figure 3:**
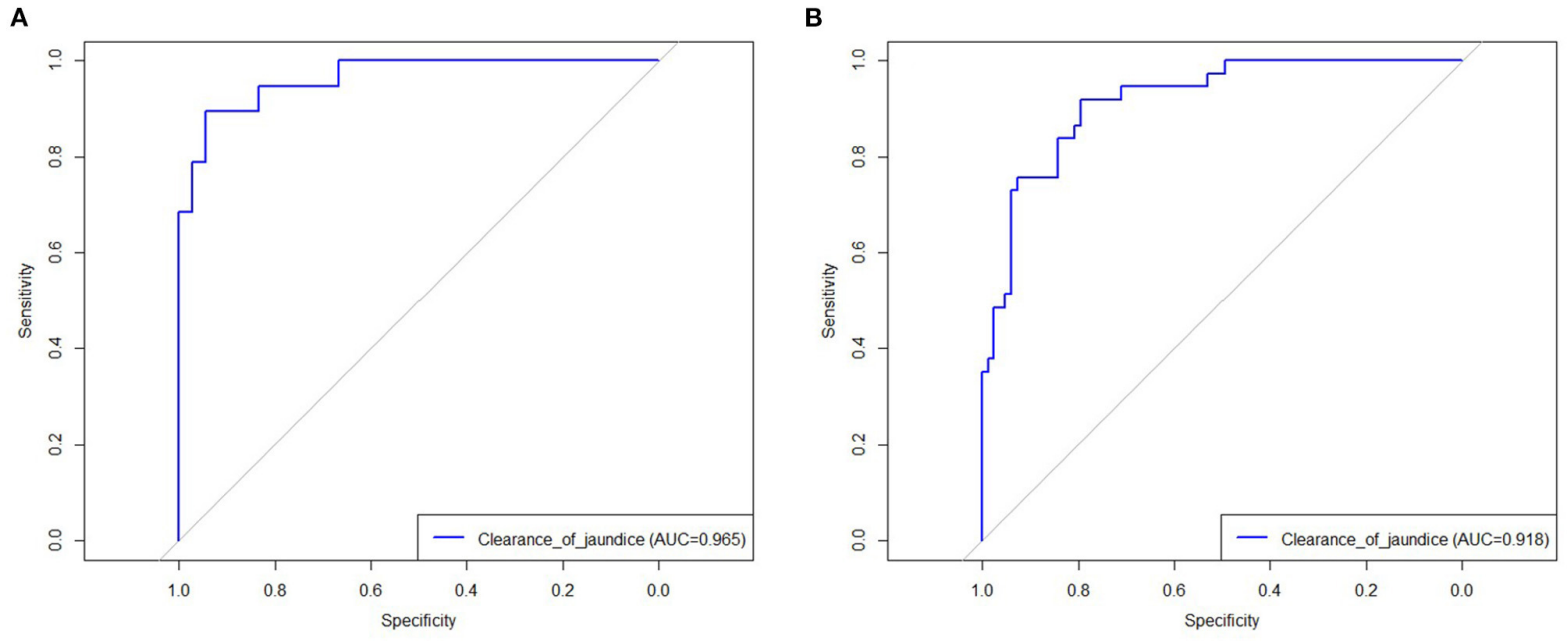
The receive operating characteristic of established model in training **(A)** and testing cohort **(B)**.

The results of the multiple logistic regression model were presented in Table 3. CMV IgM +ve associated BA (OR = 3.38; 95% CI 1.01–11.32; *P* = 0.04), ln (GGT) (OR = 0.41; 95% CI 0.22–0.80; *P* = 0.009), thickness of the fibrous portal plate (OR = 0.45; 95% CI 0.27–0.76; *P* = 0.003), LSM (OR = 1.19; 95% CI 1.06–1.34; *P* = 0.002), and multiple episodes of cholangitis (OR = 1.65; 95% CI 1.13–2.41; *P* = 0.01) were identified as independent predictors for CJ post-KP, which was used to construct the clinical nomogram model (Figure 2). Based on the area under the ROC curve (Figure 3), the AUC of the nomogram in the training and testing cohorts were 0.96 and 0.91, respectively.

**Table 3:** Multivariate logistic regression analysis to construct nomogram in training cohort.

**Variables**	**Coefficients**	**OR**	**95% CI of OR**	***P*-value**
CMV IgM + ve associated BA	1.22	3.38	1.01–11.32	0.04
Surgery days	−0.01	0.98	0.95–0.12	0.53
In (GGT)	−0.88	0.41	0.22–0.80	0.009
Thickness of fibrous portal plate	−0.80	0.45	0.27–0.76	0.003
LSM	0.18	1.19	1.06–1.34	0.002
Metavir score	0.73	2.08	0.66–6.59	0.21
Multiple episodes of cholangitis	0.50	1.65	1.13–2.41	0.01

*CMV, cytomegalovirus; BA, biliary atresia; GGT, γ-glutamyl transpeptidase; LSM, liver stiffness measurement*.

## Discussion

Post-KP CJ is crucial for the prognosis of BA patients. Several studies have indicated that an unsuccessful KP reduces the NLS time and requires early liver transplantation (6, 12). Wang et al. reported that a successful KP enabled patients to achieve long-term event-free survival and a rapid CJ rate within 4 weeks that exhibited a good predictive value (6). Additionally, Ge et al. found that uncleared post-KP jaundice was a risk factor for a shorter NLS time and earlier liver transplantation (12). In this study, we found that several clinical factors, including CMV IgM +ve associated BA, ln (GGT), thickness of the fibrous portal plate, LSM value, and multiple episodes of cholangitis, were associated with the status of CJ in BA patients post-KP. We then constructed a visual nomogram model using logistical regression analysis to accurately predict CJ in BA patients post-KP. Further, the AUC of the nomogram revealed a robust predictive performance in both the training and testing cohorts, which may help clinicians to predict CJ more accurately and aid in clinical decision-making.

Our results showed that the rates of CMV IgM +ve associated Davenport classification in the uncleared jaundice group were significantly higher than those in the cleared jaundice group in both the training and testing cohorts. Further, the results of multivariate logistic regression revealed that it was an independent risk factor for uncleared jaundice, which was similar to the results of previous studies. Shen et al. found that BA patients with CMV infection were associated with a lower rate of CJ and a higher possibility of cholangitis (22). Zhao et al. also reported that Biliary atresia patients who were also infected with CMV had a poorer prognosis, particularly with respect to jaundice clearance (23). Additionally, Zani et al. showed that infants with CMV IgM +ve Davenport classification were always associated with more jaundiced, worse liver function, and a greater degree of inflammation and fibrosis in histology compared with CMV IgM-ve isolated BA (24). They believed that CMV IgM +ve BA often led to a reduced rate of JC, shorter NLS time, and higher mortality, which is consistent with our study.

We revealed that the preoperative GGT level of the uncleared jaundice group was significantly lower than that of the cleared jaundice group, and our multivariate analysis results suggested that patients with a lower preoperative level of GGT were more inclined to develop post-KP failure CJ. Some studies also have investigated the relationship between preoperative GGT levels and the outcome of the KP. Sun et al. found that the preoperative GGT level in BA patients who survived for >5 years with normal liver function was significantly higher than in those who died from liver failure within a year post- KP (25). Besides, Shankar et al. retrospectively analyzed 113 BA patients and found that 12.3% of patients with preoperative GGT <200 IU/L experienced a shorter time from the KP to liver transplantation and poorer transplant-free survival than those with preoperative GGT > 200 IU/L (26). These results seemed to suggest that a low preoperative level of GGT may be associated with poor prognosis after KP. However, no exact results were proposed to explain it. Besides, limited sample size and retrospective data collection may also contribute to this result. Further studies may be needed.

As a non-invasive tool, the abdominal ultrasound has been widely used to help evaluate infants for BA (27), but few studies investigated the correlation between ultrasound signs and CJ post-KP. This study emphasized the importance of the thickness of the fibrous portal plate and LSM value, which were collected by ultrasound for this novel prediction model. Additionally, we investigated the correlation between the thickness of the fibrous portal plate and CJ. Our results suggested that a thicker fibrous portal plate was related to higher rates of CJ. It is well known that sufficient bile flow through the bile capillary is essential for the achievement of CJ post-KP. Good bile flow often leads to a rapid reduction in bilirubin levels in BA patients. A thicker fibrous portal plate may contain more bile capillaries, which may contribute to sufficient flow of bile and a higher possibility of CJ. Additionally, the LSM value mainly reflects the degree of liver fibrosis, which is one of the main pathological features of BA (28). Previous studies have applied the LSM assessment for the diagnostic and prognostic prediction of BA (29–31). Wu et al. found that an LSM value > 7.7 kPa was a predictive factor for discriminating BA patients from other cholestatic patients, and patients with an LSM value > 16 kPa often require early liver transplantation (29). Liu et al. also showed that the LSM value at 3 months was associated with 2-years NLS post- KP (31). In our study, the preoperative LSM values in the uncleared jaundice group were significantly higher. A higher LSM value is always suggestive of more severe inflammatory fibrosis (28), this progressive inflammatory fibrosis often deteriorates the injury narrowing the bile duct (32), which may contribute to a poor CJ rate.

It has been recognized that postoperative recurrent cholangitis is a critical clinical factor that leads to the failure of the KP, a shorter NLS, and the requirement for earlier liver transplantation (33, 34). Our study also suggested that cholangitis was an independent risk factor of successful CJ. Therefore, cholangitis was a significant portion of prediction model.

Although many studies have indicated several risk factors associated with the short- and long-term outcomes of BA patients post-KP (17, 35, 36), there are few established visual predictive models to determine its prognosis. Liu et al. constructed a nomogram to predict the 2-year NLS in BA patients post-KP and showed good performance in a validation cohort (31). However, there was a difference compared with the results of our study. The clinical factors they included were not completely consistent with those of our study, the thickness of the fibrous portal plate and times of cholangitis were not included in their study. The majority of the clinical factors they included were postoperative, but the clinical factors selected in our study were mainly preoperative. Thus, we aimed to screen high-risk patients who may require more frequent follow-ups and early preparation of liver transplantation. Additionally, the clinical factors of our model were all accessible by regular non-invasive examinations, which may improve the feasibility of clinical practice.

There are several limitations to our study. First, this was a single-center retrospective study, which may reduce the generalizability of our results. Second, the sample size of this study was small. Finally, the performance of the established models may be affected by the lack of multicenter external validation. Therefore, further studies with larger sample sizes and multicenter external validation are required. Despite these limitations, we firstly investigated that preoperative thickness of the fibrous portal plate and LSM value measured by ultrasound were related to CJ post-KP. Besides, this is the first study to establish a visual nomogram to predict CJ post-KP and to present good predictive performance. In the context of precision medicine, this model may help clinical decision-making and improve the management of BA patients.

## Conclusion

Our nomogram model including several clinical factors effectively predicts CJ in patients post-KP, which could aid in clinical decision-making.

## Data Availability Statement

The original contributions presented in the study are included in the article/supplementary material, further inquiries can be directed to the corresponding author/s.

## Ethics Statement

The study was approved by the Ethics Committee of the West China Hospital of Sichuan University. Written informed consent was obtained from the patients' parents, according to the provisions of the Declaration of Helsinki.

## Author Contributions

YZ: data curation, formal analysis, and writing–original draft. QW: methodology and writing–original draft. SP: data curation, resources, and software. JW: data curation and formal analysis. BX: methodology. JL: conceptualization and data curation. SJ: conceptualization, funding acquisition, supervision, and writing–review and editing. All authors contributed to the article and approved the submitted version.

## Funding

This study was supported by a grant from the National Natural Science Foundation of China (81571473).

## Conflict of Interest

The authors declare that the research was conducted in the absence of any commercial or financial relationships that could be construed as a potential conflict of interest.

## Publisher's Note

All claims expressed in this article are solely those of the authors and do not necessarily represent those of their affiliated organizations, or those of the publisher, the editors and the reviewers. Any product that may be evaluated in this article, or claim that may be made by its manufacturer, is not guaranteed or endorsed by the publisher.

## Abbreviations

BA: biliary atresia
NLS: native liver survival
CJ: clearance of jaundice
KP: Kasai procedure
CMV: cytomegalovirus
TBIL: total bilirubin
ALT: alanine aminotransferase
AST: aspartate aminotransferase
SA: serum albumin
ALP: alkaline phosphatase
TBA: total bile acid
GGT: γ-glutamyl transpeptidase
INR: international normalized ratio
PT: prothrombin time
LSM: liver stiffness measurement
ROC: receiver operating characteristics curve
AUC: area under the receiver operating curve.
